# Limited transfer and retention of locomotor adaptations from virtual reality obstacle avoidance to the physical world

**DOI:** 10.1038/s41598-022-24085-w

**Published:** 2022-11-16

**Authors:** Anika Weber, Ulrich Hartmann, Julian Werth, Gaspar Epro, John Seeley, Peter Nickel, Kiros Karamanidis

**Affiliations:** 1 Department of Mathematics and Technology, University of Applied Sciences Koblenz, Joseph-Rovan-Allee 2, 53424 Remagen, Germany; 2 Sport and Exercise Science Research Centre, School of Applied Sciences, London South Bank University, London, SE1 0AA UK; 3 Institute for Occupational Safety and Health of the German Social Accident Insurance (IFA), 53757 Sankt Augustin, Germany

**Keywords:** Motor control, Sensorimotor processing, Occupational health, Public health

## Abstract

Locomotor training based in virtual reality (VR) is promising for motor skill learning, with transfer of VR skills in turn required to benefit daily life locomotion. This study aimed to assess whether VR-adapted obstacle avoidance can be transferred to a physical obstacle and whether such transfer is retained after 1 week. Thirty-two young adults were randomly divided between two groups. A control group (CG) merely walked on a treadmill and an intervention group (IG) trained crossing 50 suddenly-appearing virtual obstacles. Both groups crossed three physical obstacles (transfer task) immediately after training (T1) and 1 week later (T2, transfer retention). Repeated practice in VR led to a decrease in toe clearance along with greater ankle plantarflexion and knee extension. IG participants crossed physical obstacles with a lower toe clearance compared to CG but revealed significantly higher values compared to the VR condition. VR adaptation was fully retained over 1 week. For physical obstacle avoidance there were differences between toe clearance of the third obstacle at T1 and the first obstacle at T2, indicating only partial transfer retention. We suggest that perception–action coupling, and thus sensorimotor coordination, may differ between VR and the physical world, potentially limiting retained transfer between conditions.

## Introduction

Given uneven ground, slippery surfaces, or obstacles blocking the way, walking in everyday life is somewhat challenging. In this context, the neuromotor system must be able to adapt its motor strategies to cope with such external variations. Falls and their physical^1^ and economic^2^ consequences may be reduced if means can be found to enhance locomotor skills through training. Perturbation-based balance training aims to reduce the number of falls and resulting severe consequences through participant experience of repeated, unexpected slip- or trip-like perturbations to gait, enhancing predominately reactive balance response^3,4^. These paradigms incorporating perturbation-based balance training have been used for many years to improve postural control mechanisms (e.g. increasing base of support) and reduce the likelihood of falls^4,5^. In contrast to perturbation-based balance training which focuses on reactive locomotor adaptations during tripping, obstacle avoidance training is about avoiding tripping as such and thus addresses protective mechanism in human gait. It has been shown that humans adjust their locomotor commands with repeated practice of obstacles avoidance and use a lower toe clearance aimed at reducing active musculature in the lower extremity^6,7^ and hence potentially reduce muscle mechanical work at a cost of a lower safety margin. Moreover, it has been shown that these adaptive changes at the lower extremity can be partly transferred to the untrained leg^6,8^. However, the methods used for the assessment and training paradigms targeting adaptations and transfer of locomotor skills usually require complex mechanical devices that may not only be expensive but also restrict use to dedicated locations. An alternative paradigm mitigating the complexity of instrumentation mentioned above relies on perturbations induced using virtual reality (VR). Research has previously shown that VR-based training using visual perturbations can produce compensatory adaptations that prevent injuries due to slips, trips, and falls, without the need for other perturbation devices (see^9^ for a review). Use of visual perturbations (e.g. tilting the virtual environment) has led to adaptations in spatiotemporal gait parameters^10–12^, muscle activity and kinematic responses^10^. Those findings indicate a promising effect of VR-based training of adaptation of stability control.

Effective learning and adaptation is often associated with characteristics of transfer and retention. Previous studies of mechanical slip- and trip-perturbations have shown a partial retention over several weeks or even years, whereas demonstrating transfer has been more challenging (e.g.^4,13–15^). Skill transfer may be particularly challenging for VR-based training as learned skills have to be transferred between quite different worlds (virtual and physical). It is known that egocentric distance judgments are limited in virtual environments (see^16,17^ for reviews) potentially leading to different perception–action coupling, which would impede the transfer to the physical world. Until now, few studies have investigated transfer and retention of VR skills for gait and balance training. Parijat and colleagues^18^ demonstrated that slip-like compensatory movements (both proactive and reactive) learned by tilting virtual environments can be applied to physical world conditions. Similarly, VR-based obstacle avoidance training studies found that improved skills can, at least partially, be transferred from virtual to physical obstacles^19,20^. However, no transfer was found from one leg to the other^21^. To our knowledge, only one study investigated retained transfer of learned skills from virtual- to physical world contexts^19^, and this over a short 24 h period. In this study participants performed 40 VR-based obstacle avoidance trials before retention to physical obstacles was assessed. As retained transfer was tested after VR retention it cannot be excluded that the additional practice trials affected outcome measures for the physical obstacles.

The purpose of our study was to examine whether and to what extent a learned locomotor skill can be transferred from virtual to physical environments and whether such skills are retained in virtual and physical environments over 1 week. We used the paradigm of training obstacle crossing during treadmill walking in order to assess locomotor adaptation, transfer and retention phenomena in highly controlled and reproducible tasks. We hypothesized that a single session of VR-based obstacle avoidance training would lead to adaptation in locomotor behavior (i.e. lower obstacle toe clearance and changes in joint kinematics), that these refinements would be transferred to a physical obstacle condition and at least partially retained for VR and physical obstacle conditions.

## Methods

### Participants and experimental design

Thirty-two healthy young adults (sixteen males, sixteen females; age 22.7 ± 1.8 years; height 177 ± 10 cm; mass 73.5 ± 11.5 kg) voluntarily participated in the present study after providing their written informed consent. To address the current investigation of adaptation, retention and transfer in physiologically and neurologically healthy young participants, they were screened for inclusion criteria via a questionnaire, i.e. normal or corrected-to-normal vision (glasses or contact lenses) and absence of known or diagnosed neurological and musculoskeletal impairments. Only young healthy participants were included to mitigate at best any bias caused via sensorimotor disfunctions or diseases on the outcome of our study. The study was approved by the ethics committee of the University of Applied Sciences, Koblenz and met all requirements for human experimentation in accordance with the Declaration of Helsinki^22^.

**Figure 1:**
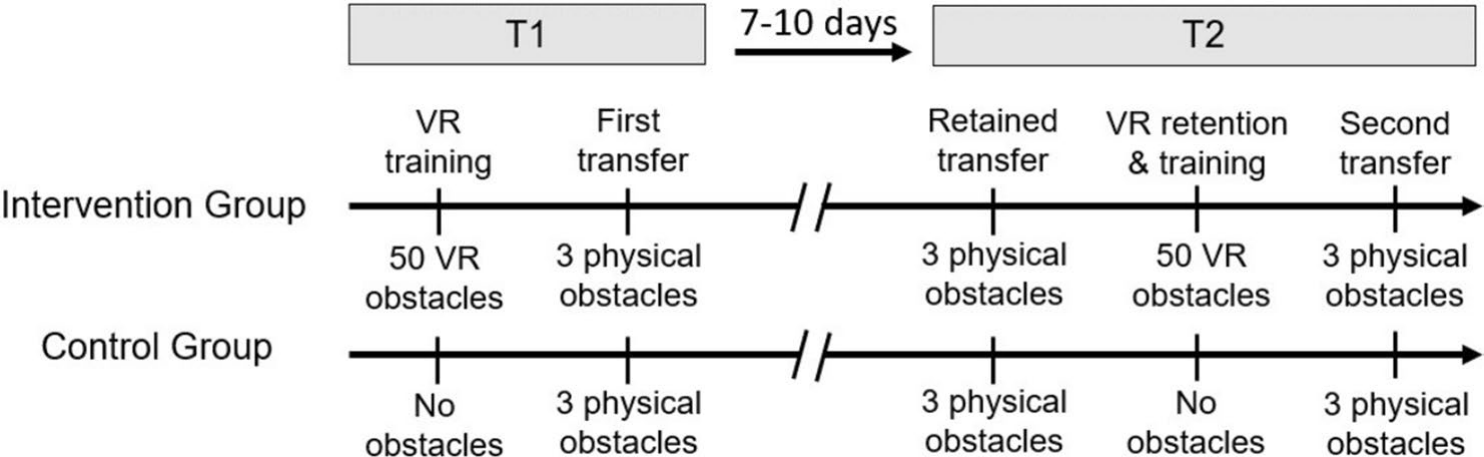
Experimental protocol illustrating the days of measurement (T1 session 1; T2 session 2), tasks within sessions and type and number of obstacles for the two groups.

Our participants were balanced in gender and pseudo-randomly assigned by study personnel using MS Excel (RAND function) into two groups: a control group, CG (eight males, eight females; 22.9 ± 1.7 years; 177 ± 9 cm; 74.4 ± 10.4 kg) and an intervention group, IG (eight males, eight females; 22.4 ± 1.9 years; 177 ± 10 cm; 73.5 ± 11.5 kg). All training and testing involved participants walking on a treadmill (pulsar, h/p/cosmos, Nussdorf-Traunstein, Germany) and having to avoid virtual and/or physical obstacles. The protocol consisted of two laboratory visits 7–10 days apart (see Fig. 1). For safety reasons, all participants wore a harness attached to the arch of the treadmill for the entirety of training and testing. Participants experienced no other exposure to virtual or physical obstacles between the first and second training but were allowed to continue with their normal physical activities.

### Experimental procedure and data acquisition

Kinematics of the two obstacle avoidance tasks were recorded using an eight-camera optical motion capture system (120 Hz; Oqus 7/Oqus 5, Qualisys, Gothenburg, Sweden). A 50-marker full-body model was used to determine each subject’s locomotor responses to obstacle avoidance. Markers were placed additionally on the head-mounted display (HMD, four markers, VIVE Pro, HTC Corporation, Taoyuan, Taiwan), on the physical obstacle made of polyurethane foam (five markers) and on the treadmill (four markers) and were used for data acquisition and analysis. Unity software (Version 2019.2.7f2, Unity Technologies, San Francisco, CA, USA) was used to create the virtual environment, in which participants saw an endless corridor, a geometrically accurate model of the treadmill and its handrails, the virtual obstacles, a feedback scale (only for IG), and their own body in outline form^21^. The body avatar was updated dynamically using live marker position data.

To control the suddenly-appearing physical obstacles, a custom-built wireless controlled electromagnetic device was fixed to an aluminum profile in front of the treadmill. A polyurethane foam obstacle (height 10 cm × depth 10 cm × width 30 cm) was held and released electromagnetically by means of a flat piece of ferromagnetic metal attached to the top of the obstacle. Another flat piece of metal (15 cm × 15 cm × 0.1 cm) attached to the bottom of the obstacle prevented it from rolling over. The foam obstacle was reattached manually to the reactivated electromagnet to repeat the obstacle avoidance task.

**Figure 2:**
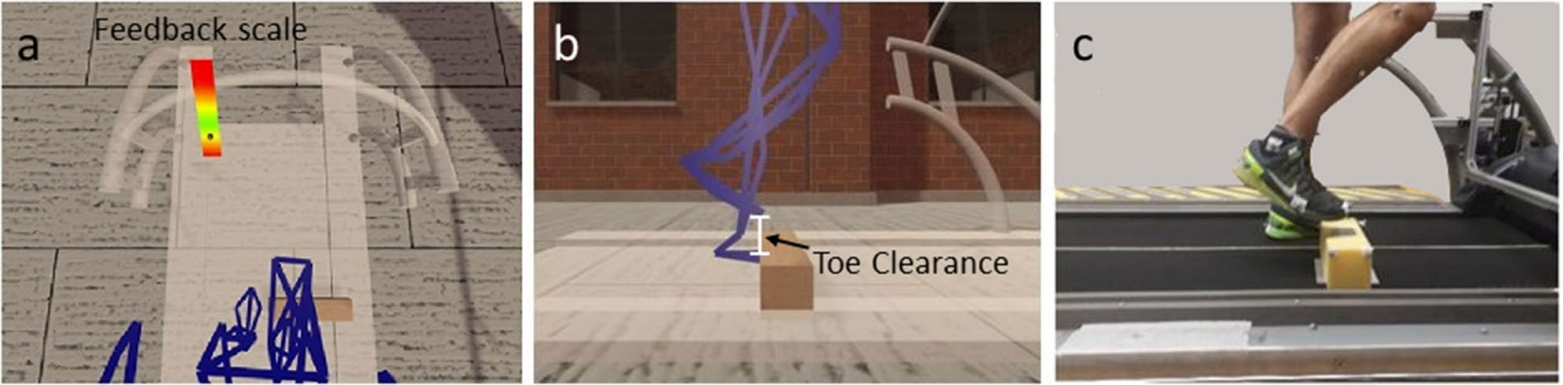
(**a**) Virtual environment containing a corridor, a model of the treadmill, avatar of the participant, virtual obstacle and visual feedback about avoidance height. The black open circle shows the position of the target area. (**b**) Participant avoiding the virtual obstacle. (**c**) Participant avoiding the physical obstacle.

For the first training session (T1), all participants (IG and CG) were familiarized with the set-up for about 12 min in a three-part procedure. They walked on the treadmill (1) without wearing the HMD, (2) whilst wearing the HMD and holding the treadmill handrails, and (3) whilst wearing the HMD having let go of the handrails. For all tasks, treadmill walking velocity was set to 1.3 m/s. The participants of the IG had to avoid 50 unilateral virtual obstacles (height 10 cm × depth 10 cm × width 50 cm) appearing 80 cm in front of their right leg at touch-down of that leg (detected via synchronized motion capture). This distance was chosen so that it did not affect rhythmic walking cadence. The obstacles appeared at random times which were fixed in the same sequence for all participants and for both trainings. Participants received visual feedback about their toe clearance height directly after each avoidance. As they were instructed to cross the obstacle within a given target range, feedback was provided in the form of a gradient color scale located at a fixed position in the virtual environment (Fig. 2a). An open black circle indicated the clearance height of the participant’s toe above the front edge of the virtual obstacle and remained in that position until the next obstacle was crossed. Participants were given two instructions. Firstly, they were asked to adapt their toe clearance to a target height in the lower yellow range (position of the black open circle in Fig. 2a), which corresponded to 3–5 cm above the leading edge of the virtual obstacle. Secondly, they were asked to avoid toe clearance below the target height, i.e. clearance in the lower red range, which corresponded to less than the safety margin of 3 cm. The participants received a detailed explanation of the target area before the training, using a picture of the scale. However, they were not informed about the meaning of the color scale. They only knew that they should cross the obstacle in the lower yellow range and not below it, and that the lower end of the scale meant that they would hit the obstacle. CG participants walked through the obstacle-free virtual corridor for the same duration as the IG.

After the VR-based training in T1 all participants had to cross three physical obstacles with the same leg as in the virtual reality training to test for transfer from virtual to physical obstacle conditions (first transfer). Prior to the transfer test all participants walked obstacle-free for four minutes on the treadmill without HMD to allow re-acclimation to the physical environment. The physical foam obstacles were of the same height and depth as those in VR (Fig. 2b) and of width 30 cm (Fig. 2c). They were triggered, unpredictably for participants, by their right heel touchdown. The physical obstacle was located at the same distance (± 2.5 cm) as in the virtual condition. Participants were instructed to cross each physical obstacle as low as possible. No feedback was given about the distance between toe and obstacle in order to test the transfer of the skills acquired in the virtual environment. They were informed beforehand that the obstacles were made of foam and therefore collisions at any time would not pose risk of injury.

All participants in training session 2 (T2; 7–10 days after T1; Fig. 1) had to cross three physical obstacles followed by a repetition of their protocol from T1 in order to assess retention effects and to determine whether obstacle avoidance adaptations in the virtual environment led to retained transfer to the physical obstacle. The repetition involved a VR-based familiarization prior to IG and CG training in VR (second training; retention), followed by another three trials with physical obstacles (second transfer).

### Data processing

The three-dimensional coordinates of markers from motion capture were filtered using a low-pass second-order zero-phase Butterworth filter with a 12 Hz cut-off frequency. Toe clearance was calculated as the difference between the height of the toe marker and the height of the obstacle when that marker was above the leading edge of the obstacle^21^ (Fig. 2b). Sagittal plane hip, knee and ankle angles of the right leg were calculated for the swing phase which was defined as the time between take-off and touchdown of the right foot. Lower extremity joint kinematics were analyzed over the entire swing phase of the crossing leg in order to identify the time course of any joint-related adaptive phenomena including changes in coordination. Foot take-off and touchdown were determined using the foot contact algorithms of Maiwald et al.^23^. Take-off is specified as either the local maximum of the vertical acceleration or the minimum vertical position of the toe marker. Touchdown is defined as the local maximum in the vertical acceleration curve of the heel or fifth metatarsal marker within an approximation interval based on the earlier of the two events. Hip angle was calculated from the hip center, hip joint center (calculated according to^24^) and knee joint center. Knee angle was calculated from the hip joint center, knee joint center and ankle joint center. Ankle angle was calculated from the knee joint center, ankle joint center and mid foot. All calculations were performed using customized routines written in MATLAB (version 9.3.0, The Mathworks Inc, Natick, MA, USA).

### Statistics

Four participants (two IG and two CG) were excluded from the analysis due to technical issues during the measurements (connection losses to the computer from both the wireless VR device and the wireless physical obstacle release system). Participant response adaptations to repeated practice while avoiding virtual obstacles were examined by pooling trials. Accordingly, trial data for hip, knee and ankle joint angles as well as for toe clearance were combined for obstacles 1–3 and 48–50, named *early* and *late adaptation* respectively. Statistical Parametric Mapping *t* tests (SPM^25^) were used to detect potential VR-based effects of obstacle avoidance training on sagittal plane hip, knee and ankle joint angles of the obstacle avoiding leg during swing phase. For this purpose, all analyzed kinematic trajectories were time-normalized to the swing phase (from take-off to touchdown of the right leg). All data were tested for normal distribution using the Shapiro–Wilk test (p > 0.05). Paired *t* tests were used to compare early and late adaptation of the IG for both T1 and T2. Possible retention effects for the VR condition were examined by comparing toe clearance and joint angle kinematics of the IG between late T1 and early T2 using a paired *t* test and its SPM equivalent. Furthermore, an equivalence test (two one-sided *t* test^26^, 90% confidence interval with δ = 0.1) was performed on toe clearance data from late T1 and early T2. Concerning avoiding physical obstacles for the IG and CG, the three trials for each time point (T1 and T2) were considered separately by two-sample *t* tests for toe clearance and SPM for joint angle kinematics to analyze potential transfer and transfer retention effects for the physical obstacle condition. Possible physical obstacle transfer effects were further examined by comparing the data of toe clearance late adaptation VR with first physical obstacle at T1 of the IG using a paired sample *t* test. Potential adaptations due to repeated crossing of physical obstacles were analyzed using separate paired sample *t* tests for both groups, comparing the first and third obstacles at T1. Retention of transfer in the IG was further analyzed by comparing the first and third physical obstacle at T1 with the first obstacle at T2 using a paired sample *t* test. Statistical analyses were performed either using SPSS Statistics (version 27, IBM, Armonk, NY, USA) or open-source code SPM1d (version M.0.4.8, http://www.spm1d.org) in MATLAB (version 9.3.0, The Mathworks Inc, Natick, MA, USA), with α set at 0.05. The one-sample equivalence test was performed using MS Excel (Microsoft, Redmond, WA, USA). All results in text are presented as mean ± SD.

## Results

### Locomotor adaptation and retention for VR-based obstacle avoidance

**Figure 3:**
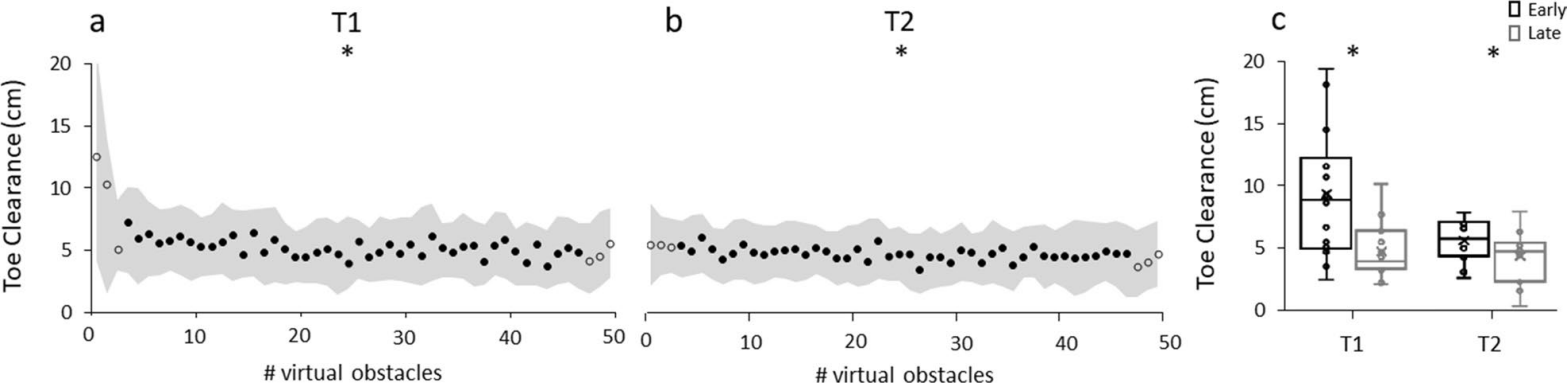
Adaptation of toe clearance for (**a**) first VR-based training (T1) and (**b**) second VR-based training (T2) for avoiding obstacles with the right leg (1–50) presented as means (circles) with standard deviations (gray shading) for all IG participants. Obstacles used to investigate adaptation (early and late) are presented as open circles. (**c**) Boxplot of early (black) and late (grey) adaptation for T1 and T2. *Significant difference between early and late adaptation (p < 0.05). No differences were detected between late T1 and early T2.

**Figure 4:**
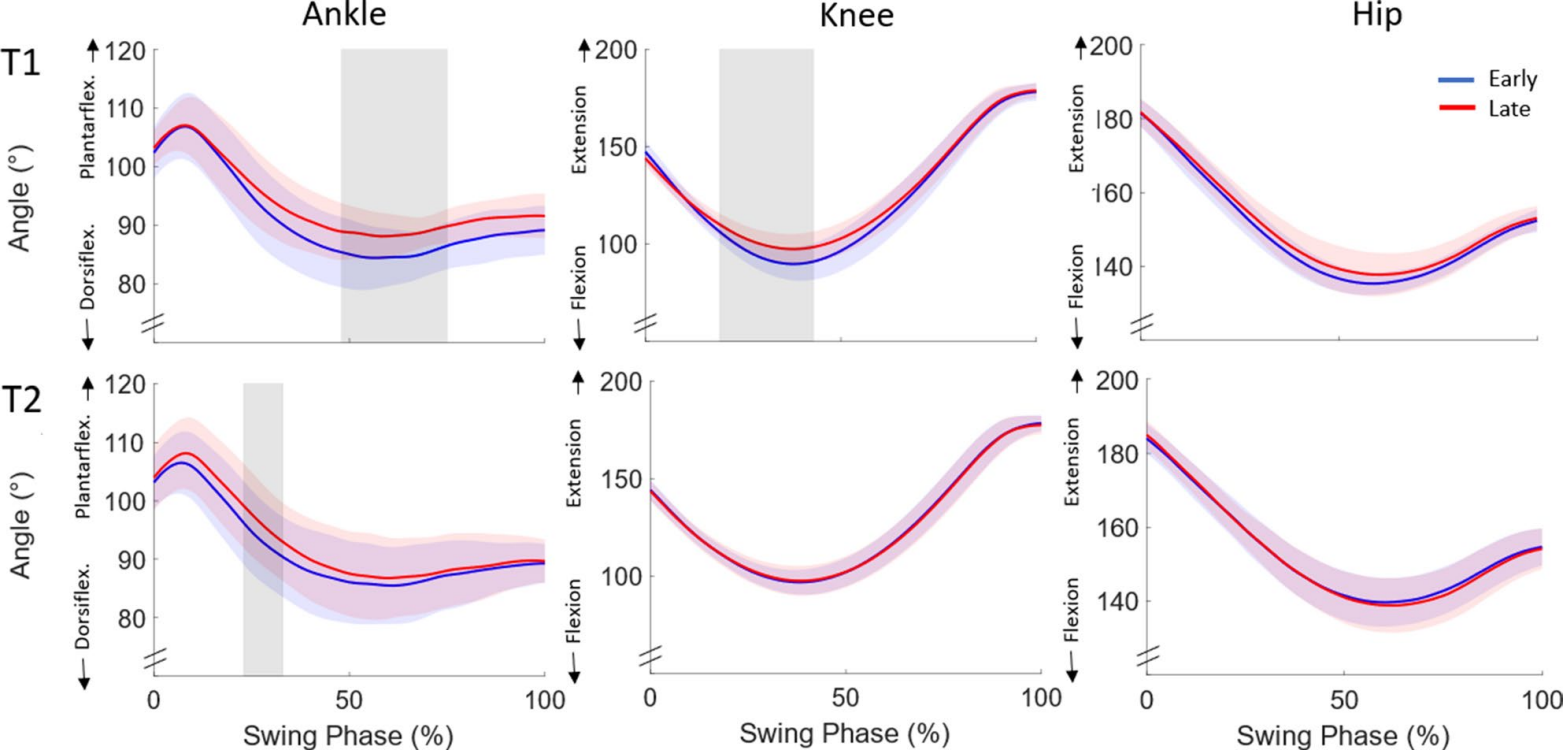
Sagittal plane ankle, knee and hip joint angle trajectories of the avoiding leg during swing phase for T1 (day 1) and T2 (post 7–10 days) for early and late adaptation as mean and standard deviation (blue and red shading) for VR obstacle avoidance. Vertical gray areas indicate significant differences between early and late adaptation.

There were no collisions between the participants’ feet and virtual obstacles during the statistically analyzed trials, but some collisions during other trials in VR training (3 participants, 7 hits in trials 10–37 at T1). The participants crossed the leading edge of obstacles (both virtual and physical) at 39 ± 5% of swing phase. Please note that similar percentages for timing were found for early and late adaptation, and in retention and transfer tasks. A significant difference (*t*(13) = 3.754, *p* = 0.002) was observed in the first training session (T1) in toe clearance between early (9.4 ± 5.0 cm) and late adaptation (4.7 ± 2.1 cm; Figs. 3a,c). Concerning the joint angle analysis using SPM for the swing phase of the avoiding right leg, the ankle joint demonstrated a significantly greater plantarflexion from 48 to 75% of swing phase (*p* < 0.001) and the knee joint a greater extension from 19 to 43% of swing phase (*p* = 0.001) for late compared to early adaptation (Fig. 4). Toe clearance decreased significantly (*t*(13) = 2.846, *p* = 0.014) from early to late phases during the second VR-based training at T2 (early 5.8 ± 1.6 cm; late 4.5 ± 1.9 cm; Fig. 3b,c). Joint angle comparison revealed a more plantarflexed ankle joint at 23–33% of swing phase for late in comparison to early adaptation for T2 (*p* = 0.020; Fig. 6).

Comparison of participants performing the task in the virtual environment at T1 and T2 showed equivalence in toe clearance (*p* < 0.001) between late adaptation at T1 (4.5 ± 1.9 cm) and early adaptation at T2 (5.8 ± 1.6 cm), though there was a trend to significant difference (*t*(13) = 2.011, *p* = 0.066). Joint angle comparisons for the swing phase of the avoiding leg between late adaptation T1 and early adaptation T2 revealed no statistically significant differences for all three joints.

### First transfer at T1, retained transfer and second transfer at T2

**Figure 5:**
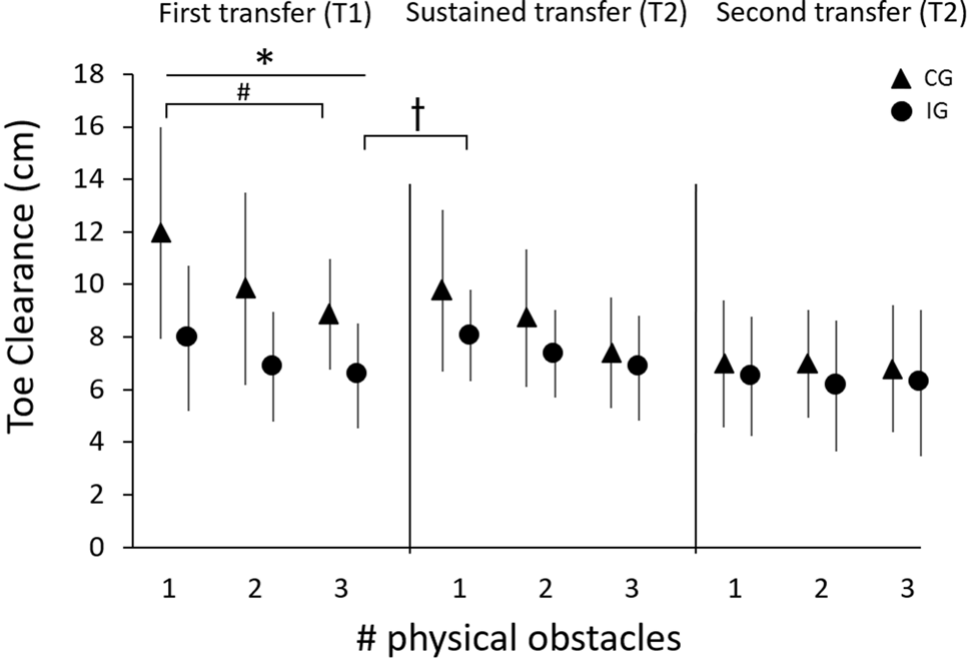
Mean and standard deviation of toe clearance for all participants of the intervention group (IG, circles; *n* = 14) and control group (CG, triangles; *n* = 14) for avoiding the three physical obstacles of each transfer task on T1 (first transfer), and T2 (retained transfer and second transfer). *Significant difference between IG and CG for obstacles 1–3 in T1 (*p* < 0.05), ♱ significant difference between third obstacle T1 and first obstacle T2 IG (*p* < 0.05), ^#^significant difference between first and third obstacle T1 CG.

**Figure 6:**
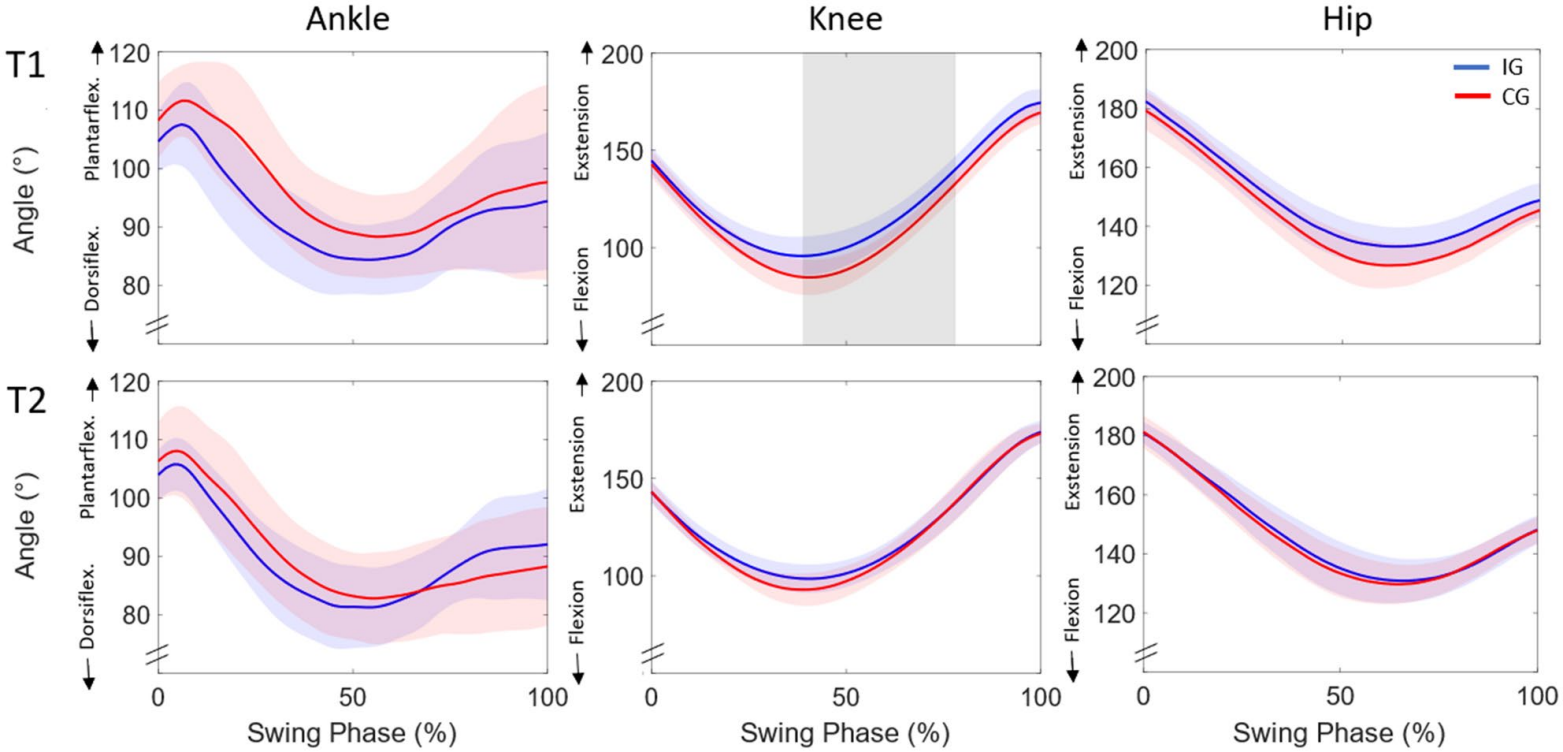
Sagittal plane ankle, knee and hip joint angle trajectories of the avoiding leg during swing phase for T1 (day 1) and T2 (post 7–10 days) for intervention group (IG) and control group (CG) as mean and standard deviation (blue and red shading) during the first physical obstacle avoidance (trial 1). The vertical gray area indicates a significant difference between groups.

Toe clearance was significantly lower in the IG compared to the CG for all three physical trials at T1 (first trial *t*(26) = 2.854, *p* = 0.008, second trial *t*(26) = 2.105, *p* = 0.047, third trial *t*(26) = 2.904, *p* = 0.007; Fig. 5). Furthermore, the IG showed a significantly more extended knee joint (for 39–78% of swing phase; *p* = 0.001; Fig. 6) while avoiding the first physical obstacle, as compared to the CG. However, when comparing late adaptation in VR at T1 with the first physical obstacle at T1 for the IG, there was a significant difference (*t*(13) = 4.014, *p* < 0.01) with higher values in toe clearance for the physical obstacle in relation to the VR condition. Within the physical trials in T1 there was no statistically significant change in toe clearance when comparing the first and third obstacle in the IG but approaching significance (*t*(13) = 1.873, *p* = 0.084). The CG significantly adapted their toe clearance from physical obstacle one to obstacle three (*t*(13) = 3.360, *p* < 0.01). Regarding the retained transfer and second transfer, no significant differences in toe clearance or joint kinematics between IG and CG were found for any of the three trials of physical obstacles. Although there were no differences between the first physical obstacle at T1 and at T2 in the IG (*t*(13) = 0.157, *p* = 0.878) we detected significant differences between the third obstacle at T1 and the first obstacle at T2 (*t*(13) = 2.429, *p* = 0.030).

## Discussion

This study investigated whether obstacle avoidance skills learned during VR-based training of young adults can be transferred to physical obstacles, and be retained over 1 week in both virtual and physical environments. We could partially confirm our hypothesis and observed limited transfer of obstacle avoidance skills from a virtual to a physical obstacle. The IG replicated similar joint angle patterns as learned in VR to cross the physical obstacle at a lower height than the CG. Furthermore, we were able to show that these adaptive refinements are partially retained over a period of 1 week for the physical obstacles since IG could retain their physical crossing performance of their first crossing at T1 but not for the third obstacle at T1 compared to T2. Altogether the outcomes give reason to question the effectiveness of VR-based training for enhancing locomotor function in physical settings over a long time period.

Results of the present study showed that participants were able to adapt their toe clearance to the target height (i.e. 3–5 cm above the virtual obstacle) and maintain that clearance (mean toe clearance in late adaptation of 4.7 cm). Our findings are thus in accordance with those of Kim and colleagues^19^ revealing similar toe clearance for the initial and final VR obstacles (initial 13 cm vs. 13 cm; final 4 cm vs. 5 cm; Kim et al.^19^ vs. our results respectively). In the current study, toe clearance significantly reduced between early and late adaptation when participants received visual feedback about their avoidance performance. It is noteworthy that participants were able to reduce their toe clearance to 5 cm when receiving feedback compared to the no-feedback value of 9 cm for our previous investigation using a similar experimental design^21^. The reduction of toe clearance is a result of combined lower knee flexion and ankle dorsiflexion for obstacle crossing. The knee joint is used to adapt the toe clearance in the first phase of obstacle crossing until the toe is above the front edge; the ankle joint is used more in the late phase of obstacle crossing (after crossing the front edge of the obstacle). Accordingly different joints may play roles in different aspects of locomotor learning during repeated obstacle crossing. Our results therefore suggest that adaptive refinements in toe clearance over repeated virtual obstacle trials are achieved by recalibrating motor task execution predominately at the ankle and knee joints and less at the hip.

The acquired avoidance patterns were partially transferred to a physical obstacle. Comparing joint kinematics between groups, significantly lower knee flexion was found for the IG compared to CG in avoiding the physical obstacle. Although descriptive differences in ankle dorsiflexion were observed between the two groups, no significant differences were found on account of the high variation amongst participants (see SD values in Fig. 6), which may be caused by a forefoot instead of heel-strike pattern for some group members. Nevertheless, the differences in knee flexion led to significantly lower toe clearance when avoiding the first physical obstacle in the IG. Despite robust adaptations in the virtual environment, transfer of VR skills to physical obstacles seems to be only partial. Participants crossed the first physical obstacle with a toe clearance of 8 cm instead of the 5 cm learned in the virtual environment. As the obstacle avoidance task was practiced in a safe virtual environment, colliding with an obstacle could not have severe consequences, i.e. participants could not trip over VR obstacles. Due to the safer environment, the participants may therefore have taken a greater risk when avoiding virtual obstacles than they did with physical obstacles^27^. IG participants, however, adapted their toe clearance within the first three performed physical obstacle trials from 7.9 to 6.5 cm on average (approaching significance with *p* = 0.084). Consequently, our results suggest that VR obstacle avoidance training leads to partial acute transfer of skills to the physical obstacle.

After 1 week without training, participants returned to the laboratory to be tested for retention of transfer (physical obstacle avoidance) and VR-based adaptation. Participants were partially able to retain their learned physical obstacle skills over 1 week. As stated above, the IG adapted their toe clearance within the first three physical trials at T1 and were able to retain this performance partially at T2 (similar performance to the first physical trial but not to the third trial at T1). As participants of the CG also adapted their toe clearance according to the absolute values (trial 1, 11.9 cm vs. trial 3, 8.8 cm on average) during the physical trials at T1 and retained this performance at T2 (trial 1 T2, 9.7 cm on average), there were no differences between groups over all three trials. The participants of the IG retained their toe clearance for physical obstacle avoidance from the first obstacle but not from the third obstacle at T1 to the first physical obstacle at T2. Since the CG was able to retain its performance from the third obstacle for 1 week, but the IG returned to a level similar to the first obstacle, we can only conclude that there was partial retained transfer. Despite limited retained transfer for physical obstacles in the IG, the participants were able to fully retain their performance in the virtual environment. Their toe clearance was not statistically significantly higher in the first three VR-based obstacle avoiding trials at T2 compared with the last three trials at T1. These differences between physical and virtual performance may be due to different perception of dimensions between virtual and real-world conditions. Regarding this, several previous studies mentioned that discrepancies between perception in real and virtual environments are contributing to distances in virtual environments being underestimated by 50–80%^16^ and heights are overestimated^28^. Since the reasons for the differences in distance estimates are multifactorial (e.g. technical factors, compositional factors, and human factors^16^), it is difficult to counteract the different perceptions by changing software parameters. Further research is needed here. Our findings indicate that perception–action coupling and hence sensorimotor coordination in virtual environments may differ from those in the physical world, potentially limiting transfer and retention and hence the effectiveness of VR-based training.

Within the second VR-based obstacle avoidance training a small but significant improvement in toe clearance was found when comparing early T2 with late T2. This improvement resulted from lower ankle dorsiflexion in early swing phase for late adaptation. However, the adaptation in toe clearance was relatively small (on average 1 cm) indicating that a steady state and ceiling effect had already occurred at T1. When testing for a second transfer to physical obstacle avoidance no further improvements were found relative to the three trials (retained transfer) performed prior to the second VR-based training. We may argue that there was no further, practically-relevant skill improvement to be transferred. On a different note, there seemed to be a threshold of approximately 6 cm toe clearance beyond which no participant progressed in the physical obstacle condition, despite adjustments between trials. There was clear skill retention for the virtual environment but only partial retained transfer and second transfer for physical obstacles. We can thus infer relatively long-lasting adaptive refinements in motor task execution strategy, though these are condition specific.

We must acknowledge that our current protocol has some limitations. In the current investigation we did not perform a pre-training baseline test in the physical obstacle condition. We chose to avoid this due to concerns about rapid learning effects potentially affecting our conclusions for transfer. Although all participants in both groups (IG and CG) were young and healthy and showed no between-group differences in age, gender, or anthropometric characteristics, we cannot rule out the possibility that obstacle avoidance at baseline differed between groups. However, since the IG showed performance differences between late adaptation in VR and the first physical obstacle, we have no reason to suggest that this limitation affects our main conclusion. Furthermore, the physical obstacle avoidance led to rapid adjustments in toe clearance within a few trials for both groups. Therefore it is not possible to distinguish clearly between the physical avoidance performance at T2 of the IG being due to partially retained transfer of the VR-based training or a partial retention of the physical avoidance skills learned at T1. In addition, it is to be noted that there were differences in instructions and setup between virtual and physical obstacle avoidance tasks. Participants were able to focus on the physical obstacle because it was constantly visible before release, whereas virtual obstacles appeared suddenly in the virtual environment. Also, participants were instructed to cross virtual obstacles in the target range whereas they were instructed to cross the physical obstacles “as low as possible.” These differences may have contributed to the diminished transfer. In this study, the participants were trained on a treadmill with obstacles of the same height to use a lower toe clearance when crossing the obstacles. In everyday life, crossing with a lower as opposed to a higher toe clearance poses a higher risk of tripping. Our goal, however, was not to reduce the risk of tripping or to replicate real-world conditions, but to use the paradigm of obstacle avoidance and reduced toe clearance as a general means of testing adaptation, transfer, and retention of VR training.

In conclusion, our findings revealed that participants in VR-based obstacle avoidance training were able to adapt their toe clearance to a target height through changes in ankle and knee joint angles in a situation in which they received visual feedback about their performance. They were able to retain those skills fully in the virtual environment over 1 week, but showed only limited transfer and retention of those skills over 1 week to avoidance of physical obstacles. Additional VR-based training did not further improve virtual-to-physical-environment transfer. It may be concluded that perception–action coupling, and thus sensorimotor coordination, in the virtual environment differs from that in the physical world, potentially inhibiting retained transfer between conditions. Accordingly, VR-based locomotor skill training paradigms need to be considered carefully if they are to replace training in the physical world.

## Data Availability

The datasets generated during and/or analyzed during the current study are available from the corresponding author on reasonable request.
